# Exploring the link between parvovirus B19 and encephalitis: a systematic review and comprehensive meta-analysis of molecular and serological evidence

**DOI:** 10.1186/s12985-025-02630-z

**Published:** 2025-02-19

**Authors:** Kashmi Sharma, Rekha Khandia, Rohan Shrivastava, Ram K. Nema, Somesh Mishra, Rupinder K. Kanwar, Ashwin A. Raut, Amit Agrawal, Vandana Gupta, Megha K. Pandey

**Affiliations:** 1 Department of Translational Medicine, AIIMS Bhopal, Bhopal, India; 2 Department of Genetics, Barkatullah University, Bhopal, MP India; 3 NIREH, Bhopal, MP India; 4 NIHSAD, Bhopal, MP India; 5 Department of Neurosurgery, AIIMS Bhopal, Bhopal, India; 6 Department of Microbiology, NDVSU, Jabalpur, MP India

**Keywords:** Parvovirus B19, Encephalitis, Prevalence, Molecular diagnostics, Meta-analysis, Neurological infection

## Abstract

Encephalitis, a severe brain inflammation, can arise due to various infectious agents, including viruses like Parvovirus B19 (B19V). Previously linked to mild neonatal and young one’s illnesses and some haematological diseases, recent evidence associates B19V with encephalitis, with no clear prevalence and mechanisms in place. This systematic review and meta-analysis aim to determine the prevalence of B19V in cases of encephalitis, exploring variations associated with diagnostic approaches, and identifying gaps in existing research to enhance clinical comprehension and diagnostic methods. An extensive search (1994–2024) was performed through PubMed, Scopus, ScienceDirect, and Cochrane databases for research and epidemiological investigations related to B19V in cases of encephalitis. Inclusion criteria focused on studies that verified B19V using molecular (PCR, NGS) or serological (IgM/IgG) techniques in cerebrospinal fluid or serum. Data analysis was done to pool the prevalence data of included studies using a random-effects model. Heterogeneity was evaluated using I^2^ statistics. Sensitivity and meta-regression analyses were conducted to evaluate variability and the effects of moderators. A total of fourteen studies involving 3,135 encephalitis patients resulted in a combined prevalence of 3% (95% CI: 2–4%). Studies using PCR indicated a greater prevalence (3%) in comparison to ELISA (1%) and NGS (2%). A moderate level of heterogeneity (I^2^ = 57.4%) was attributed to the variability in diagnostic methods and geographic distribution. Sensitivity analyses validated strong estimates, while meta-regression revealed country as a key moderator accounting for heterogeneity. Publication bias was modest. The research indicates that B19V may be involved in certain encephalitis instances, with an overall prevalence of 3%. The differences observed in the studies emphasize the need for standardized diagnostic procedures and more extensive multicentric epidemiological research.

**Supplementary Information:**

The online version contains supplementary material available at 10.1186/s12985-025-02630-z.

## Introduction

Encephalitis is a severe, often life-threatening inflammatory disease of the brain that can result from various causes, including infections from pathogens such as viruses, bacteria, and fungi, as well as from autoimmune responses. Viral encephalitis is one of the most common forms and can present with symptoms ranging from mild discomfort, such as fever and headache, to severe neurological complications including seizures, confusion, and even coma. [1]. The disease’s impact on individuals and healthcare systems is substantial, often requiring intensive medical care and long-term rehabilitation due to lasting neurological sequelae.

Among the numerous viral agents associated with encephalitis, human parvovirus B19 (B19V) has emerged as a possible but relatively rare cause. B19V is widely recognized for its role in erythema infectiosum, or “fifth disease,” primarily in children [2], where it causes mild, self-limiting rash and fever [3, 4]. However, in recent years, B19V has been implicated in an array of clinical conditions beyond this pediatric presentation, such as hematological disorders, autoimmune phenomena, myocarditis, and various neurological conditions, raising questions about its pathogenic versatility [5, 6].

This virus is of particular concern because of its ability to infect a variety of cell types beyond erythroid progenitor cells, which are central to its well-known hematologic effects. Emerging evidence suggests that B19V can persist in various tissues, including the myocardium and central nervous system (CNS), contributing to diseases in these systems. Case reports and clinical studies linking B19V to encephalitis have increased, but the overall clinical and epidemiological significance of this association remains unclear. Given the diversity of clinical presentations, diagnostic challenges, and the relatively sporadic reporting of B19V-associated encephalitis, a comprehensive review of the existing literature is warranted [4, 7, 8].

This systematic review and meta-analysis aim to identify and evaluate the existence and effect of publication bias by using visual and statistical approaches to clarify the association between B19V and encephalitis. By aggregating and analyzing existing data, this review seeks to better understand whether B19V should be considered a significant causative factor of encephalitis, to elucidate the underlying pathogenic mechanisms, and to identify gaps in current knowledge that can guide future research. This analysis also aims to provide insights for clinicians managing cases of encephalitis, as understanding B19V’s role in this context could improve diagnostic accuracy and inform therapeutic strategies.

Even though there have been many case reports and discrete studies looking at Parvovirus B19 and its association or linkage with encephalitis, it is still not clear how common the virus is in patients who have encephalitis. Studies often use different ways to diagnose (like PCR or serology), have various criteria for including patients, and look at different groups of people, which causes results to be variable in different places and time. In addition, fewer studies make it hard to generalize the finding. A meta-analysis is useful for bringing together the available information and investigating and geniting evidence on how Parvovirus B19 relates to encephalitis. This meta-analysis will combine data from several studies to get a better idea of prevalence of Parvovirus B19 in cases of encephalitis and investigate variables associated with different outcome measures. The primary aim of this meta-analysis is to determine the pooled prevalence of Parvovirus B19 in patients who have encephalitis. Additional aims of the study are to assess how the diagnostic method (molecular compared to serological) affects prevalence figures, to evaluate the influence of possible sources of variation, such as sample size, year of publication, and diagnostic method used using meta-regression and to identify and evaluate the existence and effect of publication bias by using visual and statistical approaches.

## Materials and methods

### Study plan

This systematic review and meta-analysis were done using PRISMA guidelines [9]. The review focusses to have association evidence between Parvovirus B19 (B19V) and encephalitis. The Protocol was made for inclusion rules, search plan, data extraction, and statistical methods to be employed. A detailed protocol was registered on PROSPERO (Registration No. CRD42024606982), outlining the research question, inclusion criteria, and analysis plan prior to the literature search. This protocol was followed to ensure transparency and reproducibility.

### Literature search approach

A comprehensive and extensive literature search was done to find relevant studies on Parvovirus B19 and encephalitis. The databases/search engines checked were PubMed, Scopus, Science Direct and Cochrane.

Search was done for time period from 1994 to 2024 until 20/10/24. Keywords used were: “Parvovirus B19,” “B19V,” “encephalitis,” Boolean terms like AND, OR, NOT helped narrow down the results. Articles written in English were included.

Additional manual search was conducted to find any reference lists from relevant reviews and included articles. Additional search for grey literature like conference papers (from Google search engine and databases like Shodhganga) not published was also done to avoid publication bias. Three reviewers (MKP, AG and KS) screened titles and abstracts first then examined full texts of studies to find eligible texts.

#### Eligibility conditions

Studies which met following conditions were included for the metanalysis.

1. *Study type*: original research papers published in peer-reviewed journals that had serological or molecular data specifically detecting Human Parvovirus B19 linked with neurological symptoms or encephalitis.
2. *Population*: Subjects (Human) of any age diagnosed with encephalitis showing confirmed B19V infection via molecular methods (like PCR) or serological testing (IgM, IgG antibody testing).
3. *Study design & outcome*: Only studies showing presence of B19V DNA in Cerebrospinal fluid or brain tissue or detection of antibodies in serum or Cerebrospinal fluid matched with clinical diagnosis of encephalitis. No other type of studies was included.
4. *Publication type*: Articles from peer-reviewed journals included; no conference abstracts, editorials, reviews unless they had original data were considered.

Studies were excluded if they:

1. Did not provide original information (reviews/opinion posts).
2. Were based on animals or non-human subjects.
3. Lacked specific diagnostic findings for Parvovirus B19.
4. Sample size was less than 20.

### Data extraction

Data were pulled and summarized by two reviewers who worked separately using a set of data extraction form. The information gathered from each study included:

1. *Study details*: First author, year of publication, country where the study took place, sample size.
2. *Patient details*: Age, gender (if available), Neurological manifestation.
3. *Diagnostic methods employed*: Detection of B19V DNA in CSF or brain tissue/Serum (using PCR), serological diagnosis (IgM, IgG antibodies).
4. *Results*: proportion (percent positivity) of samples out of total number of samples tested/ Number of patients tested for Parvovirus B19 and number of positive cases.

Discrepancies in data interpretation from articles were resolved through discussion, and a third reviewer (AA) was consulted in case of any confusion.

### Risk of bias assessment

The risk of bias in the studies included was checked with The Joanna Briggs Institute Prevalence Critical Appraisal Tool [10]. This reference provided 10 questions for the assessment. However, since none of the articles complied with questions 9 and 10, they were excluded from the evaluation. This did not impact the results, as only one subgroup analysis (based on diagnostic test used) was performed in this meta-analysis due to missing data. Each study was evaluated based on how participants were chosen, their comparability, and how outcomes were measured.

### Statistical analysis

Statistical analysis for primary outcome of pooled prevalence of Parvovirus B19 in encephalitis /neurological disorders patients. To access the prevalence of Parvovirus B19, we performed a meta-analysis which is a statistical procedure used to interpret a pooled finding from numerous studies for deriving an overall summary estimate by using RStudio open-source version 2024.09.1. Prevalence from each study was calculated as the ratio of positive cases to the total patients tested. These values were combined using a Mixed-effects model to include a greater variance, to consider within studies and between studies variations, as true effects may differ. This model was primarily used because of differences in study design, diagnostic methods, and patient groups. However, a check analysis was also tried using a fixed effect and random effect model for comparative assessment. The pooled prevalence was calculated with 95% confidence intervals (CIs). A forest plot was used to depict the effect size graphically. The assessment of study heterogeneity and inconsistency was done with the I^2^ statistic, where values below 50% signifies least heterogeneity while values above 50–95% indicate least to moderate heterogeneity and values above 95% indicates significantly high differences [11]. Tau-squared (τ^2^) was also reported, which shows the level of variability between studies.

### Subgroup and sensitivity analyses

Under a given set of conditions assumed, sensitivity analyses determine how different values of an independent variables effects a dependent variable. To distinguish prevalence based on various diagnostic methods, we conducted subgroup analyses for studies utilizing PCR, NGS, and ELISA for detecting Parvovirus B19. Only diagnostic test was used as sub-group for analyses as subgrouping based on other factors was not feasible either due to lack of data or availability of single finding for many groups.

Additionally, we carried out sensitivity analyses by removing one study at a time from the meta-analysis to assess the consistency of the overall prevalence estimate and to evaluate the impact of each individual study on the results.

Other methods employed for sensitivity analysis are excluding studies with extreme heterogeneity, comparing fixed effect and random effect model for meta-analyses, analyzing sub-group specific sensitivity, excluding small studies.

### Meta-regression analysis

Meta-regression analysis was performed to find sources of variability in prevalence estimates. The following factors were considered as moderators:

1. *Sample size*: Studies with larger sample size might be more accurate due to greater statistical power.
2. Publication year: More recent studies might be using improved diagnostic methods or advancements in clinical knowledge might affect the diagnosis.
3. *Geographic* *region*: The prevalence of Parvovirus B19 might differ by location because of variations in epidemiology, healthcare systems, or diagnostic capabilities.
4. *Diagnostic method*: The detection possibility of Parvovirus B19 antigen/nucleic acid or antibody might be variable due to different sensitivity and specificity of diagnostic methods used, which may result in difference prevalence been detected.

Each factor was tested on its own in simple meta-regression models, followed by a combined model that included all factors. Results were shown as regression coefficients (β) with 95% CIs and p-values.

### Publication bias evaluation

Studies mostly on prevalence of microbes which are either considered of non-clinical importance or have non-significant findings mostly remains unpublished. As meta-analysis is based on published data hence, it may be an under or over-estimate of outcome. We assessed publication bias using visual checks and statistical tests. A funnel plot was created to show the distribution of study effect sizes (prevalence) against their standard errors. Without publication bias, studies should be evenly distributed around the combined result. An uneven funnel plot might indicate bias, especially if smaller studies show more extreme outcomes.

We applied Egger’s regression test to formally test for funnel plot unevenness. A p-value less than 0.05 suggested publication bias. The Trim and Fill method was also used to estimate the number of missing studies and adjust the combined prevalence. This method "fills" the funnel plot by adding missing studies and recalculates the overall effect size to consider the impact of publication bias.

## Results

### Data extraction and quality assessment

**Fig. 1:**
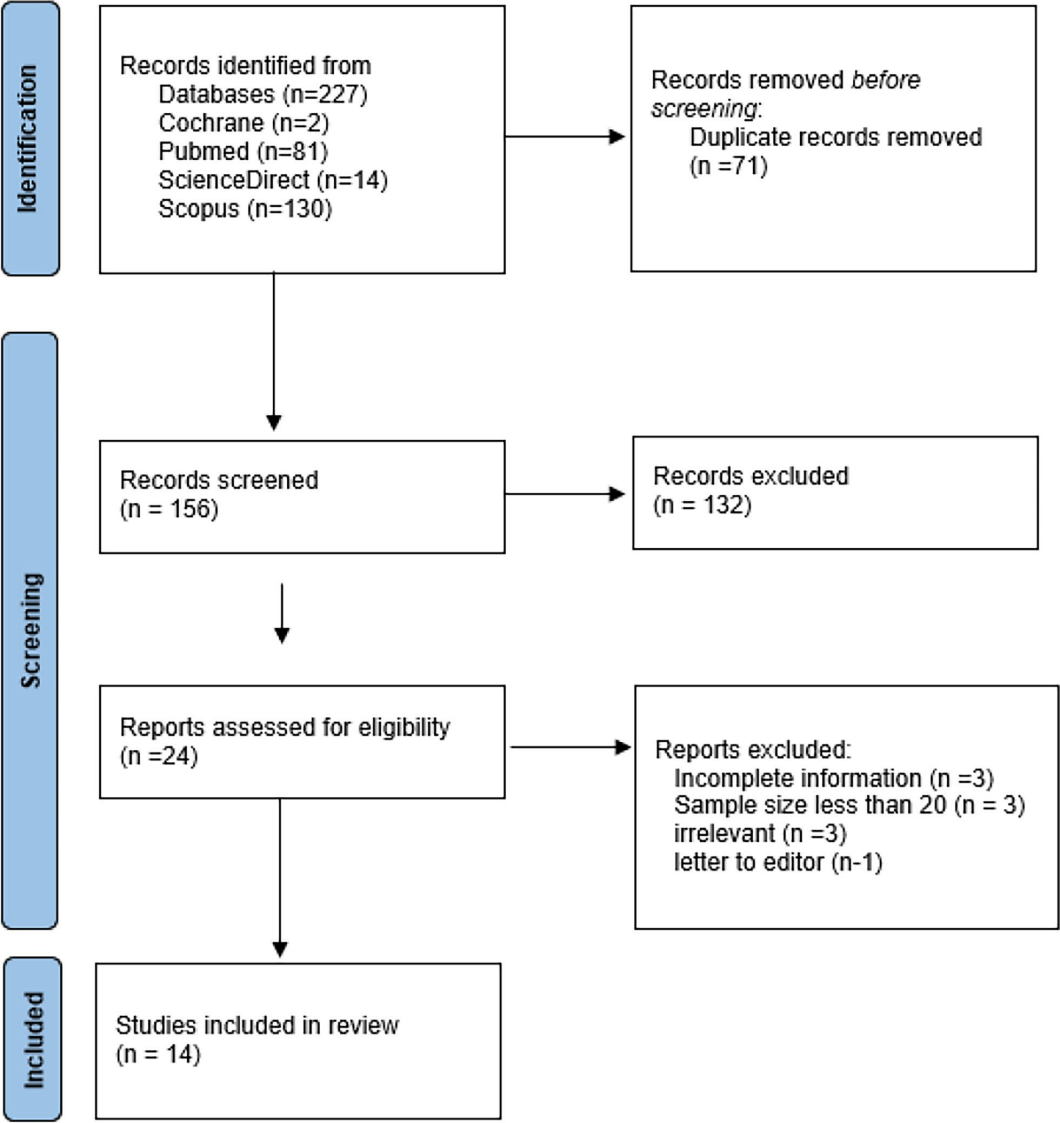
PRISMA Chart denoting the articles selection algorithm

The primary search gave a total of 227 articles across the four databases. No relevant studies could be retrieved from grey literature search. After removing duplicates (*n* = 71), 156 titles and abstracts were screened for eligibility. Of these after screening out articles, 25 full-text articles were assessed, and 11 studies were excluded for not meeting the inclusion criteria (e.g., incomplete information, irrelevant findings) (Supplementary Table: 1 and 2). Ultimately, 14 studies were included in the final meta-analysis (Fig. 1, PRISMA flowchart).

Data was extracted (Supplementary Table: 3). Total of 14 paper with 15 study rows (as one study used 2 diagnostic methods and was used as two studies) were used for data extraction. Proportion (percent positivity) of samples was calculated (number of positive cases out of total number of samples tested/ Number of patients tested for Parvovirus B19). The risk of bias in the studies included was checked with The Joanna Briggs Institute Prevalence Critical Appraisal Tool [10] (Supplementary Table 4).

Statistical analysis for primary outcome of pooled prevalence of Parvovirus B19 in encephalitis /neurological disorders patients.

The 14 included studies spanned four continents, with studies conducted in Europe, Asia, and North America and South America. The sample sizes ranged from 20 to 887, and the number of positive Parvovirus B19 cases ranged from 0 to 10.7% of the total sample in each study. The tests used for diagnosis were under 3 broad categories, i.e. PCR, ELISA and NGS. [4, 12–25].

#### Geographic distribution

Five studies were conducted in India (Dey et al., 2024; Kumar et al., 2018; Pattabiraman et al., 2022; Rathore et al., 2022; Sonowal et al., 2024), three in Italy (Monticelli et al., 2018; Parisi et al., 2016), one in London (Barah et al., 2001) and the remaining studies were spread across the United States, Brazil, Japan, China and Poland. The variation in geographic representation underscores the potential for regional epidemiological differences in Parvovirus B19 prevalence and the possible impact of diagnostic capabilities in different settings.

#### Diagnostic methods

Except one study done on serum samples [26] (all other studies were based on CSF sample testing. Most studies used PCR to detect Parvovirus B19 DNA in patient samples, typically in cerebrospinal fluid (CSF) (11 out of 15 total studies). Two studies used serological methods (ELISA) to detect IgM antibodies [12, 26] indicating recent Parvovirus B19 infection. Notably, serological methods showed a lower positivity rate compared to PCR, which may be due to the heterogeneity in sample types (one in CSF and one in Serum) and diagnostic thresholds of ELISA used. The use of IgM/ ELISA which traces the recent infection and may differ in sensitivity may have contributed to this difference.

### Pooled prevalence and subgroup analysis

**Fig. 2:**
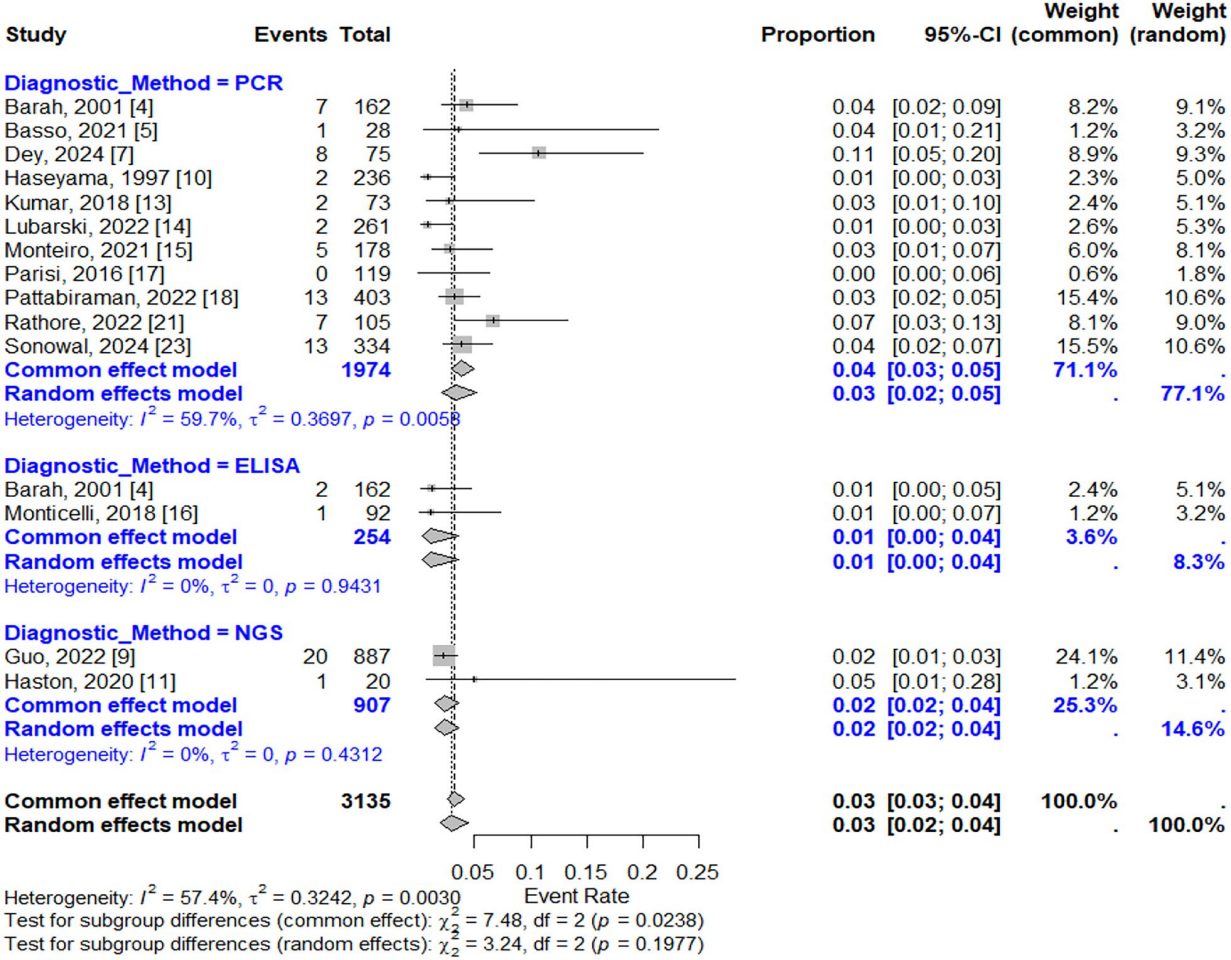
Forest Plot with 14 studies included and the subgroup analysis based on diagnostic tests. The effect sizes (proportions) from individual studies have been shown as squares with size of square box showing weight of the study and horizontal line denotes the CI. The overall pooled estimate from common effect and Random Effect model has been shown as Diamonds

The pooled prevalence of Parvovirus B19 across all studies was 3% (95% CI: 2–4%) based on the random-effects model and 3% (95% CI: 3–4%) based on the common-effects model. The forest plot (Fig. 2) visually represents the effect sizes from individual studies and the overall pooled estimate. The forest plot depicted the subgroup analysis based on diagnostic tests used. The pooled proportion based on PCR as diagnostic test gave an overall value of 3% which was higher as compared to ELISA subgroup (1.00%) and NGS ((2.00%).

The prevalence estimates varied widely across studies, with the highest reported prevalence of 10.7% in the study by Dey et al. (2024) [15] from India and the lowest at 0.0% in the study by Parisi et al. (2016) [13] from Italy. Heterogeneity among the studies was significant (I^2^ = 57.4%, *p* < 0.01), indicating substantial variability in the effect sizes, which may be due to differences in diagnostic methods, patient populations, or study design. Most of the heterogenicity was attributed to subgroup of Diagnostic method- PCR as most of the pooled prevalence was derived from those studies.

This value measures the percentage of total variation across studies attributable to heterogeneity rather than chance, which indicates a moderate to high level of variability between the studies. About 57% of the observed differences in virus positivity rates could be due to true heterogeneity among studies rather than random error. This also supports the use of a mixed-effects model, as it accounts for variability across studies in the pooled prevalence estimate.

The I^2^ values for each subgroup of different diagnostic methods is as follows. PCR: I^2^ = 59.7%; ELISA: I^2^ = 0% and NGS: I^2^ = 0%. The high I^2^ value in PCR group shows a lot of heterogeneity in studies using PCR. This difference in PCR subgroup may come from various PCR types (Multiplex, conventional, nested and real time PCR), different protocols, populations, or study conditions. The 0% I^2^ values for both ELISA and NGS indicate no significant heterogeneity. with very little variation between them.

The tau-squared (τ^2^) values calculated as ‘0’ for subgroup NGS and ELISA indicates little variation between studies within these subgroups, showing that studies using the same diagnostic method (ELISA, or NGS) had similar virus positivity rates. However, in this particular meta-analysis this could not be explained due to fewer studies in these sub-groups. However, τ^2^ = 0.3697 in PCR group suggests that about 37% of heterogenicity in PCR sub-group is accounted for due to reasons other than random sampling error.

### Sensitivity analysis

Sensitivity analyses revealed that the overall prevalence estimate was robust, with minimal changes in pooled prevalence when individual studies were excluded. Subgroup analyses indicated that studies using PCR for detection reported higher prevalence rates (3%) compared to studies using ELISA (1%) or NGS (2%). However, this difference did not reach statistical significance, likely due to the small number of studies using ELISA or NGS.

**Fig. 3:**
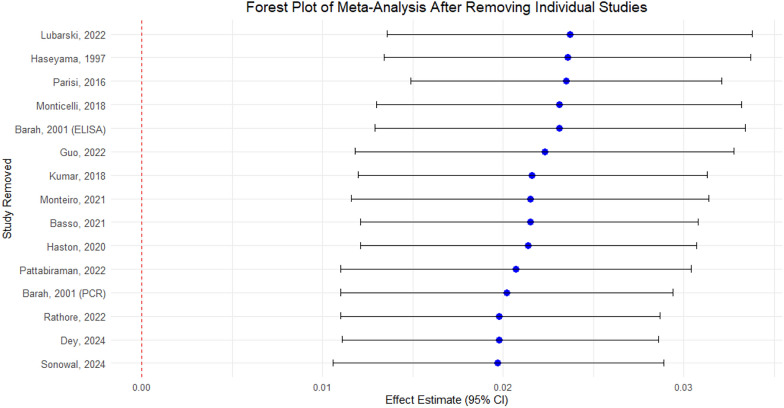
Forest Plot with Sensitivity analysis after Leave-one-out method

Sensitivity analysis after Leave-one-out method was used to make a forest plot (Fig. 3), The pooled effect estimates remain significant across all analyses (ranging from approximately 0.0197 to 0.0237). Confidence intervals did not include 0 (none of the CI touched the line of null effect), confirming a robust association. The estimates were all clustered within a narrow range, suggesting that no single study could influence the overall effect disproportionately. This forest plot supports the robustness of the meta-analysis results and highlights that while some studies contribute more to heterogeneity, their exclusion does not drastically affect the pooled prevalence.

### Meta-regression analysis

Moderate heterogeneity was detected among the included studies, as evidenced by the I^2^ statistic of 59.7% in subgroup based meta-analysis as shown in Fig. 2. This suggests that the prevalence of Parvovirus B19 in encephalitis patients varies widely across different settings and study designs. While meta-regression analysis explored potential sources of this heterogeneity, none of the examined covariates—sample size, publication year, or geographic region—were found to significantly influence effect sizes.

On individual analysis of best fit model for individual covariates following were the results based on Regression coefficients (Supplementary Table 5).

1. Geographical region Country as a moderator was checked by mixed effect meta-regression model. However, there we no significant moderation by the country variable. The pooled effect size remained consistent across all countries.
2. Publication year—Publication year was checked by mixed effect meta-regression model. There was no meaningful trend over the time. Year as a moderator did not significantly affect the effect size.
3. *Sample size*: Sample size as a moderator was checked by mixed effect meta-regression model. No significant variation in pooled effect size was found on using sample size as moderator.
4. *Diagnostic method*: Diagnostic method as a moderator was used in mixed effect meta-regression model. No significant variation in pooled effect size was found on using different diagnostic method as moderator.

Further analysis by the combined effects of multiple moderators (sample size, year of publication, country, and diagnostic method) on the variability in effect sizes across 15 studies was assessed by Mixed-Effects Model which Combines fixed effects (overall pooled effect size and moderator effects) and random effects (heterogeneity across studies). With this model, sample size, diagnostic method and publication year were not found to be significant predictors of variability in effect size, however country was found to be a significant moderator overall, with significant effects for UK and Japan. Hence most of the heterogeneity was explained by country (76.30%) and combined moderators explains for about 80.71% of heterogenicity in effect sizes. ((Supplementary Table 6).

To explain further due to moderate heterogeneity as detected among the included studies, as evidenced by the I^2^ statistic of 57.4% in subgroup based meta-analysis contributed by PCR diagnostic group, we compared the heterogeneity metrics such as I^2^, τ^2^ and R2 with and without PCR as reference (Supplementary Table 7). We found that heterogeneity exists within PCR subgroup but is not due to diagnostic method itself. When analyzed along with other diagnostic methods, heterogeneity is fully explained by sampling variability (I2 = 0%). The inclusion of publication year and diagnostic method as moderators did not explain the observed heterogeneity within PCR subgroup.

**Fig. 4:**
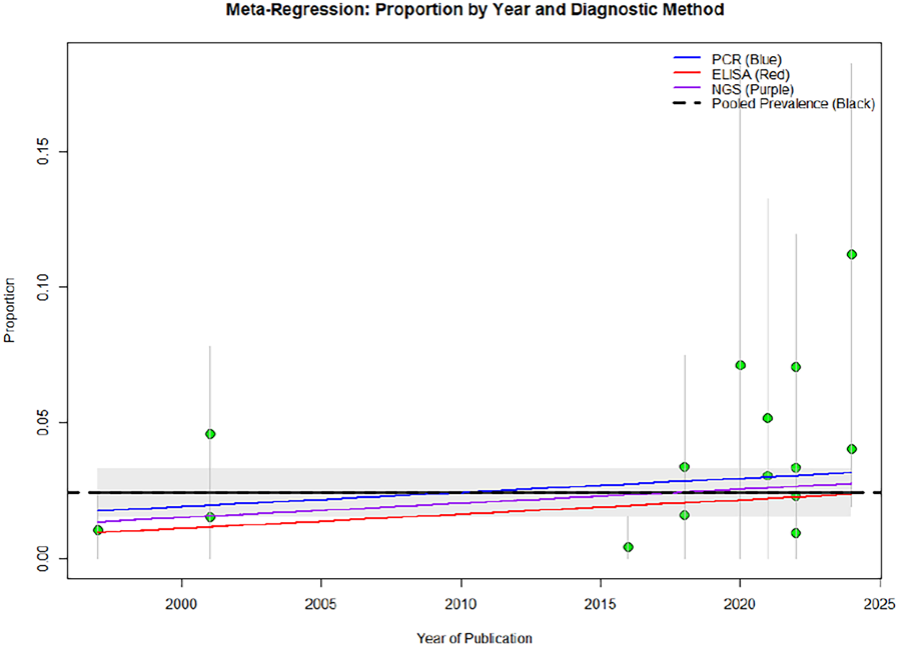
A meta-regression plot with proportion (prevalence from individual studies on Y-axis and Year of publication on X-axis). Trend lines for PCR, ELISA and NGS with pooled prevalence line shown in blue, red, purple and black color

A meta-regression plot was created with proportion (prevalence from individual studies on Y-axis and Year of publication on X-axis). Trend lines for indicating specific diagnostic method for PCR, ELISA and NGS with pooled prevalence line were also added. (Fig. 4). The findings from this plot denotes that publication year does not have significant effect on pooled prevalence. There are slight differences in prevalence between PCR, ELISA and NGS but are statistically non-significant, but shows a slight increasing trend with years. This also confirms the findings of the meta-regression. These finding suggests that the observed variability in prevalence rates may be driven by unmeasured factors, such as differences in diagnostic protocols, patient selection criteria, or study type etc. Slight increased trend in prevalence rate might be due to increased sensitivity of molecular methods used for screening.

### Publication bias evaluation

**Fig. 5:**
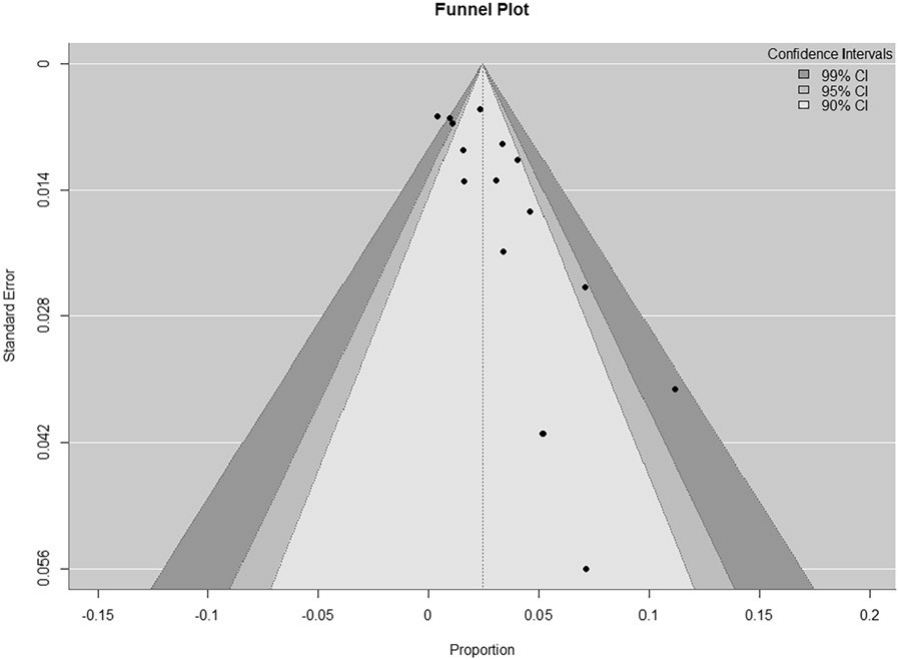
Funnel Plot for publication bias assessment. Individual dots denote each study with Proportion on X-axis and Standard Error on Y-axis

Funnel plot was made with the studies used for metanalysis (Fig. 5). Visual inspection of the funnel plot revealed symmetry suggesting that studies with smaller or insignificant results are not systematically missing from the analysis. The plot also indicates a good overall consistency of effect sizes across studies. The spread of the points, particularly at the base of the funnel, reflects the moderate-to-substantial heterogeneity observed in the meta-analysis.

**Fig. 6:**
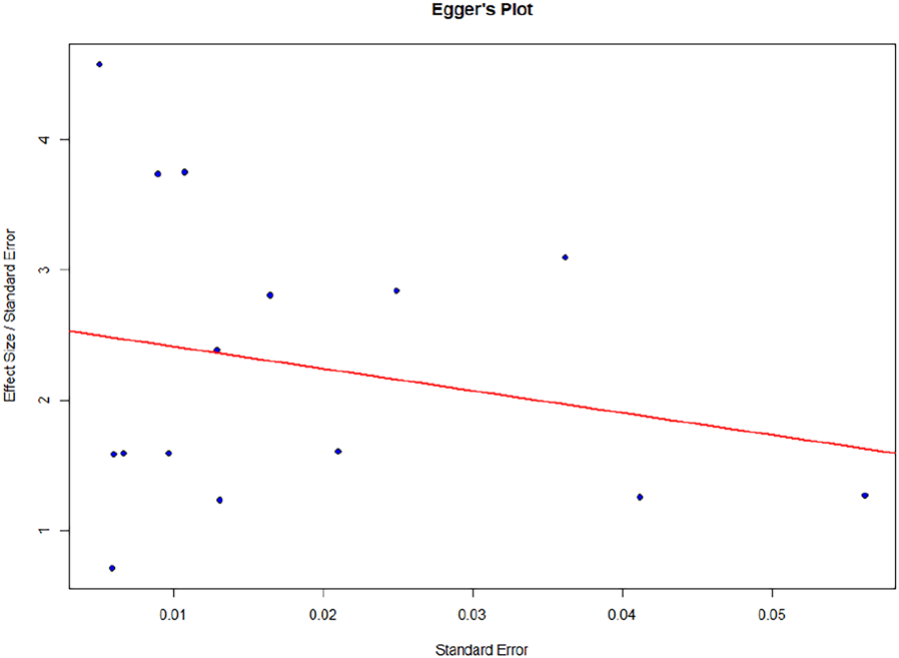
Egger’s plot to assess Funnel Plot asymmetry. Individual dots denote each study with Pro Standard Error on X-axis Effect Size/Standard Error on Y-axis. Mild Slope in regression line denotes asymmetry in the funnel plot

**Fig. 7:**
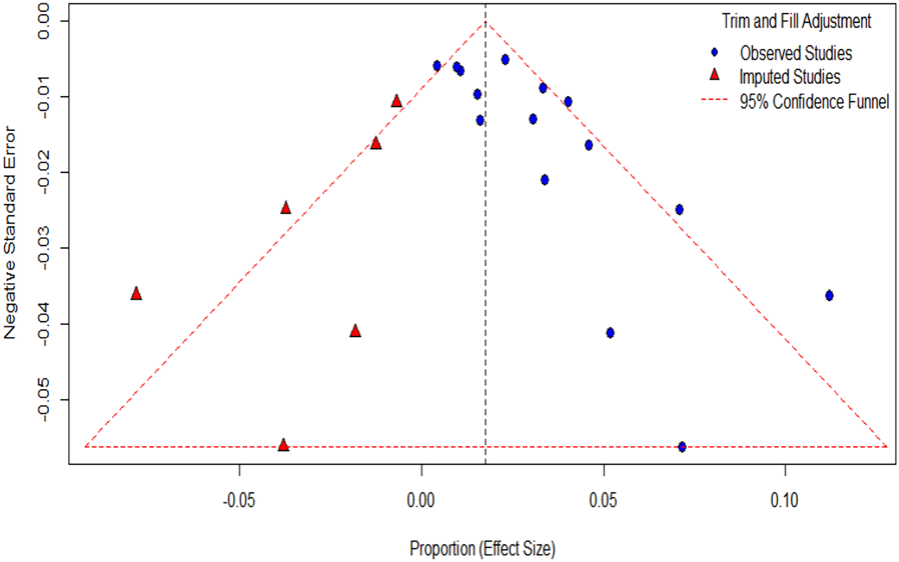
Inverted Funnel plot with Trim and Fill method with confidence interval Individual dots denote each study with Proportion on X-axis and Standard Error on Y-axis

To further explain Egger’s plot was created (Fig. 6) which visualizes the regression used to assess potential funnel plot asymmetry, which can indicate publication bias or small-study effects in a meta-analysis. It denoted slight asymmetry, with only a mild slope in the regression line. To quantify the asymmetry, egger’s regression test for funnel plot asymmetry was done. Model used was weighted regression with multiplicative dispersion using standard error as predictor. The test gave t = 4.1204 with *p* = 0.0012, suggesting that the intercept (b = 0.0007) is significantly different from Zero indicating asymmetry in the funnel plot (Supplementary Table 8). This publication bias may be due to selective reporting of the significant findings, differences in the study quality or methodology or true heterogeneity amongst studies. However, the small intercept denotes that publication bias might be there but its impact on overall results is likely to be limited. To assess further, a trim and fill analysis was done to estimate the number of potentially missing studies and their impact. The Fig. 7 shows Funnel plot with Trim and Fill method. The Trim and Fill method identifies 3 studies missing (likely due to publication bias) on the left side of the funnel plot indicating smaller effect studies to be missing from the meta-analysis. The standard error of the estimate of number of studies (2.6402) indicates the level of uncertainty in this number. The adjusted pooled effect size is 0.0211 with a narrow confidence interval [0.0187, 0.0235]. The pooled estimate is statistically significant (*p* < 0.05) (Supplementary Table 9). After accounting for publication bias, the meta-analysis still finds a minimum effect on the pooled effect size, with no heterogeneity remaining after adjustment.

## Discussion

The growing body of evidence linking Parvovirus B19 to encephalitis specially in sporadic case reports underscores the need for clinical awareness of this condition, especially in patients with neurological symptoms and a history of recent viral infection. Early recognition and differential diagnosis could significantly affect the treatment and its outcome in this severe clinical condition. This meta-analysis aimed to synthesize the available molecular and serological evidence linking Parvovirus B19 to encephalitis. The results derived out of this meta-analysis have important clinical and research implications. Clinician’s awareness about Parvovirus B19 implication in a proportion of encephalitis cases. Most of the cases included as studies was a cross-sectional surveillance study. However, in cases of encephalitis where common etiologies (e.g., culture positive bacteria, HSV, enteroviruses) have been excluded, testing for Parvovirus B19 may be warranted. This also needs particular attention in patients with underlying hematological or immunological conditions that may predispose them to severe Parvovirus B19 infection (as seen in other studies reporting case reports or case series, which were excluded in this meta-analysis due to inclusion criteria of more than 20 samples used in the study) ([17, 27–29].

The pooled analysis of 14 studies involving 3,135 encephalitis patients yielded a pooled prevalence of 3% for Parvovirus B19, suggesting that the virus may play a role in a small proportion of encephalitis cases. This meta-analysis supports the hypothesis that Parvovirus B19 is involved in a subset of encephalitis cases, with a pooled prevalence of around 3%. However, the moderate heterogeneity between studies points to the prevalence variability depending on other factors population characteristics, diagnostic methods, and study design. Further research is essential to interlink the clinical significance of Parvovirus B19 in encephalitis, especially in differentiating active infection from past exposure.

The studies included in this meta-analysis employed a variety of diagnostic methods, with most using PCR to detect Parvovirus B19 DNA and a smaller subset using ELISA to detect viral antibodies and NGS for detecting viral DNA (two in each method). Interestingly, serological studies tended to report lower prevalence rates than those using molecular techniques. This difference could be attributed to several factors such as diagnostic sensitivity and study population differences. Molecular assays, particularly those detecting Viral DNA, may be more sensitive to detection of infection, while IgM/IgG may only detect immune reaction to virus. Moreover, except one study all were done on CSF. The kinetics of antibodies reaching to CSF could also be a factor in lesser detection of antibodies in CSF [30]. Given that Parvovirus B19 infections often resolve before the onset of neurological symptoms, serological methods may underestimate the prevalence of present infections in encephalitis patients. The included studies also varied significantly in their patient populations and geographic settings. Factors such as regional differences in Parvovirus B19 epidemiology, variations in healthcare infrastructure, and differences in the clinical criteria for encephalitis diagnosis may have contributed to the observed heterogeneity. While meta-regression analysis explored potential sources of this heterogeneity, none of the examined covariates—sample size, publication year, or geographic region—were found to significantly influence effect sizes. This finding suggests that the observed variability in prevalence rates may be driven by unmeasured factors, such as differences in diagnostic protocols, patient selection criteria, or study quality. It is also possible that the heterogeneity reflects true biological variation in Parvovirus B19’s role in encephalitis across different populations. For example, previous studies have suggested that certain genetic or immunological factors may predispose individuals to more severe manifestations of Parvovirus B19 infection, including CNS involvement. Such variability in host susceptibility could explain why some studies report higher prevalence rates than others. One of the most significant findings of this meta-analysis was the slight detection of publication bias. Publication bias is a well-known problem in clinical research, particularly in fields where negative findings are less likely to be published. In the article concerned with Parvovirus B19 and encephalitis, it is possible that studies failing to detect an association between the virus and CNS disease were either not published or not included in major databases. The funnel plot showed some asymmetry, with smaller studies reporting higher prevalence rates of Parvovirus B19. This was confirmed by Egger’s regression test, this publication bias may be due to selective reporting of the significant findings, differences in the study quality or methodology or true heterogeneity amongst studies. However, the small intercept (b = 0.0007) denotes that publication bias might be there but its impact on overall results is likely to be limited, indicating that the positive association between Parvovirus B19 and encephalitis may be inflated by selective publication of positive results. The Trim and Fill method estimated that three studies may have been omitted due to publication bias, and when these studies were imputed, the adjusted prevalence of Parvovirus B19 in encephalitis patients dropped to 2.11%. While this reduction is relatively modest, it highlights the importance of accounting for publication bias when interpreting the results of meta-analyses.

This meta-analysis represents the most comprehensive analysis to date of the molecular and serological evidence linking Parvovirus B19 to encephalitis. By pooling data from 14 studies across diverse geographic regions and patient populations, this data provides a more accurate estimate of the prevalence of Parvovirus B19 in encephalitis patients than any individual study. Second, the use of random-effects modelling allowed us to account for the significant heterogeneity between studies, providing a more robust estimate of the overall effect size. However, there are also several limitations that should be considered. The primary limitation is the heterogeneity between studies in PCR sub-group, which was not fully explained by the meta-regression analysis. As mentioned, the variability in PCR diagnostic methods (Real time PCR, conventional PCR, Digital Droplet PCR), patient populations, and clinical criteria for encephalitis likely contributed to the observed heterogeneity. The applicability of population/country (different study setting) as a moderator affecting major part of heterogeneity also explained the reason to a major extent. Hence this meta-analysis again emphasizes on the regional variation in prevalence of infections, necessitating the molecular epidemiological approach to understand many aspects of infectious diseases. Future studies should aim to standardize these variables to improve the comparability of results. Another limitation is the small number of studies included in the analysis, particularly for serological and NGS based methods. The small sample size limited the power of subgroup analyses and may have obscured potential differences in prevalence between molecular and serological diagnostic techniques.

From a research perspective, the detection of moderate heterogeneity and publication bias underscores the need for large-scale, methodologically rigorous studies to better understand the role of Parvovirus B19 in CNS infections. Future studies should be aiming to:

1.*Standardize diagnostic criteria*: Studies should adopt consistent criteria for defining encephalitis and should standardize the use of molecular versus serological methods for detecting Parvovirus B19.

2.*Increase sample sizes*: Small studies are more susceptible to bias and may overestimate the association between Parvovirus B19 and encephalitis. Larger studies are needed to provide more reliable estimates of prevalence. However, this should be also focusing on the findings of some important differential clinical findings derived from individual case reports and case series of Parvovirus B19 causing encephalitis.

3.*Address potential confounders*: Future research should account for potential confounding factors, such as patient age, immune status, and comorbidities, which may influence the likelihood of Parvovirus B19 involvement in encephalitis.

## Conclusions

The results of this meta-analysis are consistent with previous reviews that have suggested a modest association between Parvovirus B19 and encephalitis [15]. However, the overall prevalence estimates of 3% is lower than some earlier estimates, which reported prevalence rates as high as 10%.[15]. This discrepancy may be due to differences in the inclusion criteria or diagnostic methods used in the included studies. Notably, earlier studies may have relied more heavily on serological methods, which tend to report higher positivity rates than molecular techniques.

Previous systematic reviews of viral encephalitis have highlighted the importance of identifying viral etiologies, particularly in cases of encephalitis with an unknown cause. While HSV remains the most common viral cause of encephalitis, other viruses, including enteroviruses, Varicella zoster virus, and less frequently, Parvovirus B19, have been implicated. This meta-analysis adds to the growing body of evidence supporting the inclusion of Parvovirus B19 in the differential diagnosis of encephalitis, particularly in immunocompromised patients or those with hematological conditions.

## Supplementary Information

Additional file1.

## Data Availability

No datasets were generated or analysed during the current study.
