# Supplementary material for: Exploring the link between parvovirus B19 and encephalitis: a systematic review and comprehensive meta-analysis of molecular and serological evidence
Source: Virol J. 2025 Feb 19;22:42. doi: 10.1186/s12985-025-02630-z (PMC11837615; doi:10.1186/s12985-025-02630-z)
Supplement: Supplementary file 1 — Additional file1. [file 12985_2025_2630_MOESM1_ESM.docx]

**Supplementary details**

**Table 1. Search strategy**

| **Database** | **Search strategy** |
| --- | --- |
| SCOPUS | TITLE-ABS-KEY ( parvovirus AND b19 AND encephalitis ) |
| ScienceDirect | Title, abstract, keywords: parvovirus b19 encephalitis |
| COCHRANE | 2 Trials matching parvovirus b19 encephalitis in Title Abstract Keyword |
| PubMed | (("parvovirus b19, human"[MeSH Terms] OR ("parvovirus"[All Fields] AND "b19"[All Fields] AND "human"[All Fields]) OR "human parvovirus b19"[All Fields] OR ("parvovirus"[All Fields] AND "b19"[All Fields]) OR "parvovirus b19"[All Fields]) AND ("encephalities"[All Fields] OR "encephalitis"[MeSH Terms] OR "encephalitis"[All Fields])) AND (1000/1/1:2024/10/5[pdat]) |

**Table 2. Studies excluded with reasons**

| **Study author (year)** | **Reason for exclusions** |
| --- | --- |
| Takasawa et al, 2016 ^[2]^ | Letter to Editor |
| De Oliv Eira et al, 2017 ^[6]^ | Not exclusively of encephalitis, sample size<20 |
| Eltounsi et al, 2006 [8] | Irrelevant |
| Petitgas et al, 2023 ^[19]^ | Incomplete information |
| Pypa et al, 2019 ^[20]^ | Incomplete information |
| Roshdy et al, 2023 ^[22]^ | Incomplete information |
| van Lieverloo et al, 2021 ^[24]^ | Irrelevant |
| Bai et al, 2024 ^[3]^ | Irrelevant |
| Ilhan et al, 2012 ^[12]^ | sample size<20 |
| Yoto et al, 2001 ^[25]^ | sample size<20 |

Table 3 Characteristics of included studies

| **Study Author (Year)** | **Country** | **Biological material colllected** | **total samples** | **% virus positivity** | **Diagnostic method** | **Gene** | **Age** | **Sex** | **Neurological manifestation** | **Study period** | **Source** |
| --- | --- | --- | --- | --- | --- | --- | --- | --- | --- | --- | --- |
| Barah, 2001 ^[4]^ | London | CSF | 162 | 4.3  1.2 | Nested PCR  IgM ELISA | NS1 and VP1 | 1 day to 15 years and 2 months | M/F | Undiagnosed meningoencephalitis | March, 1997 to March, 1998 (outbreak) | Pubmed |
| Basso, 2021 ^[5]^ | Veneto, Italy | CSF | 28 | 3.6 | Real-time PCR | Not given | 18-64 years | M/F | Suspected central nervous system infection | 2011 to 2017 | Scopus |
| Dey, 2024 ^[7]^ | Bhubneshwar, odisha, India | CSF | 75 | 10.7 | Multiplex Real time PCR | NS 1 | 2-50 years | M/F | Acute encephalitis syndrome | March 2022-November 2023 | Pubmed |
| Guo, 2022 ^[9]^ | Zhengzhou, China | CSF | 887 | 2.25 | NGS | Not given | Not given | M/F | Encephalitis, GBS | January 2020 to October 2021 | Scopus |
| Haseyama, 1997 ^[10]^ | Sapporo, Japan | CSF | 236 | 0.8 | Nested PCR | VP1 and VP2 | Not given | M/F | Meningitis, encephalitis and encephalopathy | 1990-95 | Scopus |
| Haston, 2020 ^[11]^ | Atlanta, United states | CSF | 20 | 5 | mNGS | Not given | 6 months to 17 years | M/F | Encephalitis | April 2013 and December 2017 | Scopus |
| Kumar, 2018 ^[13]^ | Agra, India | CSF | 73 | 2.73 | Multiplex PCR | Not given | 1 month – 16 years | M/F | Acute viral encephalitis | March 2014-March 2015 | Scopus |
| Lubarski, 2022 ^[14]^ | Poland | CSF | 261 | 0.8 | PCR | Not given | 2.5 months to 18 years | M/F | Neurological autoimmune diseases | 3 January 2017 and 2 December 2019 | Scopus |
| Monteiro, 2021 ^[15]^ | Western Brazilian Amazon, Brazil | CSF | 178 | 2.8 | Real time PCR | VP1 and VP2 | <12 years | M/F | Central Nervous System (CNS) infection | 2012 and 2017 | Scopus |
| Monticelli, 2018 ^[16]^ | Italy | Serum | 92 | 1.1 | ELISA IgM and IgG | Not applicable | 18–87 years | M/F | Meningitis, encephalitis and meningoencephalitis | Jan 2004-Dec 2016 | Scopus |
| Parisi, 2016 ^[17]^ | Veneto region, North eastern Italy | CSF | 119 | 0 | Real time PCR | Not given | <14 years | M/F | Suspected central nervous system infection | Jan 2012-May 2015 | Scopus |
| Pattabiraman, 2022 ^[18]^ | West bengal, India | CSF | 403 | 3.2 | PCR | Not given | 0-45 years | M/F | Meningoencephalitis | 2016 and 2018 | Scopus |
| Rathore, 2022 ^[21]^ | Rajasthan, India | CSF | 105 | 6.67 | Multiplex real-time PCR | Not given | 1 month to 15 years | M/F | Acute febrile encephalopathy | June 2018 to January 2020 | Scopus |
| Sonowal, 2024 ^[23]^ | Assam and neighboring areas, India | CSF | 334 | 3.9 | Multiplex real time PCR | Not given | 3 months – 95 years | M/F | Acute encephalitis syndrome | January 2019 to 2022 | Scopus |

| Quality assessment of frequency studies using the Joanna Briggs Institute prevalence critical appraisal tool | | | | | | | | | | |
| --- | --- | --- | --- | --- | --- | --- | --- | --- | --- | --- |
| **Authors, Publication year** | **Q1** | **Q2** | **Q3** | **Q4** | **Q5** | **Q6** | **Q7** | **Q8** | **% YES** | **ROB** |
| Barah, 2001 | Y | Y | Y | Y | Y | Y | Y | N | 87.5 | Low |
| Basso, 2021 | Y | Y | N | Y | N | Y | Y | Y | 75 | Low |
| Dey, 2024 | Y | Y | Y | Y | Y | Y | Y | Y | 100 | No |
| Guo, 2022 | N | N | Y | Y | Y | N | Y | Y | 62.5 | Moderate |
| Haseyama, 1997 | N | N | Y | Y | N | Y | N | N | 37.5 | High |
| Haston, 2020 | N | Y | N | Y | Y | N | Y | N | 50 | Moderate |
| Kumar, 2018 | N | Y | Y | Y | Y | Y | Y | Y | 87.5 | Low |
| Lubarski, 2022 | N | N | Y | Y | Y | Y | Y | Y | 75 | Low |
| Monteiro, 2021 | N | Y | Y | Y | Y | Y | Y | Y | 87.5 | Low |
| Monticelli, 2018 | Y | Y | Y | Y | Y | Y | Y | Y | 100 | No |
| Parisi, 2016 | N | Y | Y | Y | Y | Y | Y | Y | 87.5 | Low |
| Pattabiraman, 2022 | Y | Y | Y | Y | Y | Y | Y | Y | 100 | No |
| Rathore, 2022 | N | Y | Y | Y | Y | Y | Y | Y | 87.5 | Low |
| Sonowal, 2024 | N | Y | Y | Y | Y | Y | Y | Y | 88.5 | Low |

**Table 4. Quality assessment**

Q1 Was the sample representative of the target population?

Q2 Were study participants recruited in an appropriate way?

Q3 Was the sample size adequate?

Q4 Were the study subjects and setting described in detail?

Q5 Was data analysis conducted with sufficient coverage of the identified sample?

Q6 Were objectives, standard criteria used for measurement of the condition?

Q7 Were the conditions measured reliably?

Q8 Was their appropriate statistical analysis?

**Table. 5. Individual Meta-Regression Analysis Results for Each Covariate**

| **Covariate** | **Coefficient (β)** | **95% CI Lower** | **95% CI Upper** | **p-value** | **R² (Explained Variance)** |
| --- | --- | --- | --- | --- | --- |
| **Sample Size** | 0.0007 | -0.0069 | 0.0082 | 0.8560 | 0.26% |
| **Publication Year** | 0.0435 | -0.1366 | 0.2237 | 0.6105 | 2.05% |
| **Geographic Region** | -2.2932 | -5.7314 | 1.1450 | 0.1701 | 18.41% |
| **Diagnostic Method** | -0.0880 | -12.3366 | 12.1605 | 0.9865 | 29.28% |

**Table. 6. The combined effects of multiple moderators (sample size, year of publication, country, and diagnostic method) on the variability in effect sizes across 15 studies was assessed by Mixed-Effects Model**

Meta-Regression Overview and Results

| Component | Details/Results | Interpretation |
| --- | --- | --- |
| Overview | The meta-regression explores the contribution of moderators (sample size, publication year, country, diagnostic method) to the heterogeneity in effect sizes. | Investigates whether variability in effect sizes across studies can be explained by these moderators. |

Key Outputs and Their Interpretation

6.1. Model Fit Statistics

| Statistic | Sample Size as Moderator | Publication Year as Moderator | Country as Moderator | Combined Moderators |
| --- | --- | --- | --- | --- |
| Log-Likelihood (logLik) | 30.1988 | 30.9020 | 16.9677 | 10.6933 |
| AIC | -54.3976 | -55.8041 | -15.9354 | 2.6133 |
| BIC | -52.7028 | -54.1092 | -16.4222 | -4.7511 |
| Interpretation | Lower AIC/BIC indicates a better fit. Sample size and publication year models had slightly better fits than the country model. Combined moderators provided the best overall fit. |  |  |  |

6.2. Residual Heterogeneity

| Statistic | Sample Size as Moderator | Publication Year as Moderator | Country as Moderator | Combined Moderators |
| --- | --- | --- | --- | --- |
| τ2 (variance) | 0.0002 (SE = 0.0001) | 0.0002 (SE = 0.0001) | 0.0000 (SE = 0.0001) | 0.0000 (SE = 0.0002) |
| τ (SD) | 0.0145 | 0.0135 | 0.0065 | 0.0058 |
| I2 (heterogeneity) | 76.65% | 79.44% | 20.15% | 13.16% |
| H2 | 4.28 | 4.86 | 1.25 | 1.15 |
| R2 (explained) | 0.00% | 0.00% | 76.30% | 80.71% |
| Interpretation | Sample size and publication year explain minimal heterogeneity. Country and combined models explain the majority of heterogeneity (R2>75% ) |  |  |  |

6.3. Test for Residual Heterogeneity

| Statistic | Sample Size as Moderator | Publication Year as Moderator | Country as Moderator | Combined Moderators |
| --- | --- | --- | --- | --- |
| QE (Cochran's Q Test) | QE(df = 13) = 50.1906, p<0.0001p < 0.0001p<0.0001 | QE(df = 13) = 77.1074, p<0.0001p < 0.0001p<0.0001 | QE(df = 7) = 10.9089, p=0.1426p = 0.1426p=0.1426 | QE(df = 4) = 3.5556, p=0.4695p = 0.4695p=0.4695 |
| Interpretation | Significant residual heterogeneity remains for sample size and year, but country and combined moderators account for most heterogeneity (p>0.05p > 0.05p>0.05). |  |  |  |

6.4. Test for Moderators

| Statistic | Sample Size as Moderator | Publication Year as Moderator | Country as Moderator | Combined Moderators |
| --- | --- | --- | --- | --- |
| QM (Test of Moderators) | QM(df = 1) = 0.0063, p=0.9370p = 0.9370p=0.9370 | QM(df = 1) = 1.3839, p=0.2394p = 0.2394p=0.2394 | QM(df = 7) = 19.5143, p=0.0067p = 0.0067p=0.0067 | QM(df = 10) = 28.1006, p=0.0017p = 0.0017p=0.0017 |
| Interpretation | Sample size and year are not significant moderators. Country and combined moderators significantly explain variability (p<0.01p < 0.01p<0.01). |  |  |  |

6.5. Model Coefficients

| Moderator | Estimate | p-Value | 95% CI | Interpretation |
| --- | --- | --- | --- | --- |
| Sample Size | 0.0000 | 0.9370 | [-0.0000, 0.0000] | Not significant. Sample size does not predict effect size. |
| Publication Year | 0.0006 | 0.2394 | [-0.0004, 0.0016] | Not significant. Year does not predict effect size. |
| Country (Overall) | Significant | p=0.0067p = 0.0067p=0.0067 | - | Country explains significant variability overall. |
| Country: UK | 0.1527 | 0.0411 | [0.0065, 0.2989] | Significant. UK shows higher positivity rates compared to the baseline. |
| Country: Japan | 0.1684 | 0.0500 | [0.0000, 0.3368] | Significant at p=0.05p = 0.05p=0.05. Japan shows higher positivity rates compared to the baseline. |
| Diagnostic Method (PCR) | 0.0146 | 0.2318 | [-0.0093, 0.0385] | Not significant. PCR does not differ significantly from the baseline diagnostic method (e.g., ELISA). |

Summary

| Category | Results/Insights |
| --- | --- |
| Significant Moderators | Country (significant overall, with significant effects for UK and Japan). |
| Non-Significant | Sample size, publication year, and diagnostic method were not significant predictors of variability in effect sizes. |
| Residual Heterogeneity | Most heterogeneity was explained by country (76.30%) and combined moderators (80.71%). |
| Conclusion | Sample size and publication year have minimal impact. Country explains significant variability in effect sizes. |

**Table.7. Heterogeneity Metrics with and without PCR as Reference:**

| Model | I² (Unexplained Variability) | τ² (Variance of Residuals) | R² (Explained Variability) |
| --- | --- | --- | --- |
| PCR as Reference | 94.42% | 6.69 | 5.58% |
| Without PCR as Reference | 70.72% | 5.01 | 29.28% |

Interpretation:

1. With PCR as Reference:
   - I² = 94.42%: Most of the heterogeneity remains unexplained.
   - τ² = 6.69: High residual heterogeneity indicates substantial variability across studies.
   - R² = 5.58%: Only a small proportion of the heterogeneity is explained by the diagnostic method when PCR is the reference category.
2. Without PCR as Reference:
   - I² = 70.72%: A significant portion of the heterogeneity is still unexplained, but it is notably lower than when PCR is the reference.
   - τ² = 5.01: Lower residual heterogeneity compared to the model with PCR as reference.
   - R² = 29.28%: More heterogeneity is explained when PCR is not the reference category.

**Table 8. Egger’s Regression Test for Funnel Plot Asymmetry**

| Model used | weighted regression with multiplicative dispersion |
| --- | --- |
| Predictor: | standard error |
| Test for Funnel Plot Asymmetry |  |
| t | 4.1204 |
| df | 13 |
| p | 0.0012 |
| Limit Estimate | (as sei -> 0) |
| b | 0.0007 |
| CI | -0.0102, 0.0117 |

**Table 9. Results of Trim-and-Fill Analysis**

| **Metric** | **Value** | **Interpretation** |
| --- | --- | --- |
| **Adjusted Pooled Effect Size** | 0.0211 | The pooled effect size after accounting for publication bias. |
| **Standard Error (SE) of Estimate** | 0.0012 | Indicates high precision in the adjusted effect size estimate. |
| **95% Confidence Interval (CI)** | [0.0187,0.0235] | The true effect size is likely to fall within this narrow range with 95% confidence. |
| **Number of Imputed Studies** | 3 | Three studies were imputed on the left side of the funnel plot to address asymmetry. |
| **Standard Error of Imputed Studies** | 2.6402 | Indicates variability or uncertainty in the number of imputed studies. |
| **Impact on Funnel Plot** | Symmetry restored after imputation | The addition of imputed studies corrected the asymmetry in the funnel plot, suggesting minimal bias. |
| **Heterogeneity (I^2^)** | 0% | No residual heterogeneity remains after adjustment, indicating variability is fully explained. |
